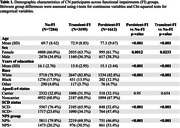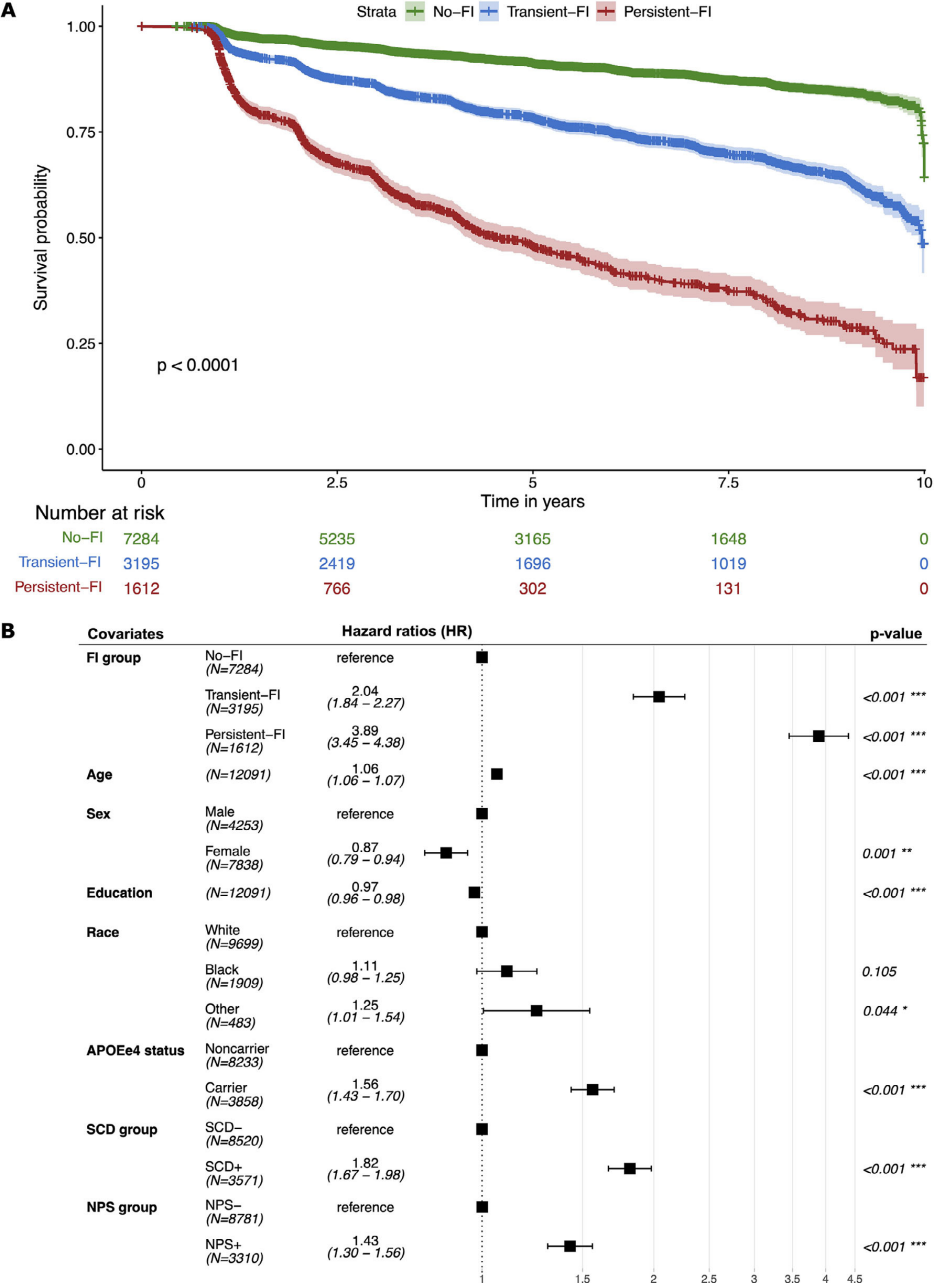# Persistent functional impairments in cognitively normal older adults and risk of cognitive decline and dementia

**DOI:** 10.1002/alz.091757

**Published:** 2025-01-09

**Authors:** Maryam Ghahremani, Zahinoor Ismail

**Affiliations:** ^1^ Hotchkiss Brain Institute, University of Calgary, Calgary, AB Canada

## Abstract

**Background:**

Maintaining functional independence is an essential aspect of healthy aging. In fact, functional dependence to perform activities of daily living (ADL) is a fundamental part of a dementia diagnosis. Newer diagnostic criteria for MCI also consider functional impairments (FI) but not to the extent of compromising functional independence. Therefore, accurate characterization of FI across the cognitive spectrum could help identify individuals who may be at a greater risk of cognitive decline and dementia, thereby improving treatment strategies. Here, we explored the utility of capturing persistent FI, in contrast to transient FI, to identify a higher‐risk group for cognitive decline and dementia.

**Method:**

Data from cognitively normal (CN) older adults from the National Alzheimer’s Coordinating Center were analyzed. From the Functional Activities Questionnaire four items were used (assembling tax records, paying bills, shopping alone, and traveling). Persistent FI was operationalized as FI present at more than two‐thirds of all study visits (TTV) prior to cognitive decline and dementia. The two comparator groups either had transient FI not meeting TTV criteria or no FI in advance of cognitive decline and dementia. Kaplan‐Meier survival curves were generated for all FI groups. Cox proportional hazard models compared incidence rates for cognitive decline and dementia across FI groups, adjusted for age, sex, education, race, APOE‐e4 status, and presence of subjective cognitive decline and neuropsychiatric symptoms.

**Results:**

The CN sample comprised 1,612 Persistent‐FI (age = 77.3±9.7; 61.7% female), 3,195 Transient‐FI (age = 72.9±8.8; 63.7% female), and 7,284 No‐FI participants (age = 69.7±8.4; 66.0% female) (Table 1). Persons with persistent FI had lower survival (p<0.0001) and a 3.89‐fold greater incidence rate for cognitive decline and dementia compared to No‐FI (CI:3.45‐4.38, p<0.001); persons with transient FI had a 2.04‐fold greater incidence rate than No‐FI (CI:1.84‐2.27, p<0.001) (Figure 1).

**Conclusions:**

CN older adults with persistent FI had greater incidence of cognitive decline and dementia than transient FI or no FI. Operationalizing FI‐related risk based on the persistence of functional impairments improves the prognostication of cognitive decline and dementia and allows for the identification of individuals who are at a greater risk in the absence of objective cognitive impairments.